# PD-1 has a unique capacity to inhibit allergen-specific human CD4^+^ T cell responses

**DOI:** 10.1038/s41598-018-31757-z

**Published:** 2018-09-10

**Authors:** Sandra Rosskopf, Beatrice Jahn-Schmid, Klaus Georg Schmetterer, Gerhard J. Zlabinger, Peter Steinberger

**Affiliations:** 1 Division of Immune Receptors and T Cell Activation, Institute of Immunology, Center for Pathophysiology, Infectiology and Immunology, Medical University of Vienna, Vienna, Austria; 2 Institute of Pathophysiology and Allergy Research, Center for Pathophysiology, Infectiology and Immunology, Medical University of Vienna, Vienna, Austria; 3 Department of Laboratory Medicine, Medical University of Vienna, Vienna, Austria; 4 Division of Clinical and Experimental Immunology, Institute of Immunology, Center for Pathophysiology, Infectiology and Immunology, Medical University of Vienna, Vienna, Austria

## Abstract

T lymphocytes have a crucial role in initiating and promoting type I allergies. Their responses are tightly regulated by numerous activating and inhibitory signals provided by APCs. Here we have addressed the role of the major coinhibitory receptors PD-1, CTLA-4, BTLA and LAG-3 in allergen-specific CD4^+^ T cell responses. PBMCs of healthy individuals and 41 patients allergic to house dust mites, birch, grass or mugwort pollen were stimulated with allergenic extracts and expression of coinhibitory receptors on responding CD4^+^ T cells was assessed. Blocking antibodies to PD-1, CTLA-4, BTLA and LAG-3 were used to evaluate the role of coinhibitory pathways. Allergen-specific CD4^+^ T cells showed strong upregulation of PD-1, LAG-3 and CTLA-4 upon stimulation, whereas BTLA was downregulated. Blockade of PD-1 strongly enhanced proliferation and cytokine production (IL-10; T_H_1 cytokines IFN-γ, TNF-α; T_H_2 cytokines IL-5, IL-13) of allergen-specific CD4^+^ T cells derived from allergic as well as non-allergic individuals. BTLA blockade enhanced proliferation but not cytokine production in response to house dust mite extract. Blocking LAG-3 was ineffective and surprisingly, we observed reduced proliferation and cytokine production in presence of a CTLA-4 antibody. Our results point to a unique potency of PD-1 pathways to dampen allergen-specific human T cells.

## Introduction

Allergen-specific CD4^+^ T cells play crucial roles in type I allergy^1–3^. T_H_2 cells and IL-4 producing T_fh_ cells promote allergy by inducing class switching to the production of IgE in B cells recognizing allergens^4^. Moreover, secretion of IL-13 and IL-5 by these cells stimulates airway epithelial cells and eosinophils, thereby promoting airway hyperreactivity and asthma^5^. Induction of allergen-specific T_reg_, which are thought to efficiently dampen T_H_2 responses, upon allergen-specific immunotherapy was reported in several studies^6–10^. T_H_1 effector T cells specific to allergens might on the one hand be beneficial by counteracting T_H_2 responses, but such cells might on the other hand significantly contribute to allergic pathologies such as delayed type hypersensitivity reactions^11^. The presence of allergen-specific CD4^+^ T cells is, however, not limited to sensitized individuals as T cells reactive to common allergen sources can be detected in the majority of healthy individuals^12,13^. It is therefore thought that the quality and magnitude of T cell responses to allergen sources will influence the development of allergies, but many aspects of this interrelation are still insufficiently understood^5,14,15^.

The response of T cells that recognize antigen is tightly regulated by numerous stimulatory and inhibitory signals. These signals are generated upon interaction of activating and inhibitory receptors with their cognate ligands expressed on antigen presenting cells (APC) and cells of surrounding tissues^16^. Signals from costimulatory receptors like CD28 are required for productive immune responses. However, inhibitory receptors expressed on T cells, often referred to as “immune checkpoints”, are important for limiting and terminating T cell responses. Engagement of the receptor PD-1 (programmed cell death protein 1) by its ligands, PD-ligand 1 and PD-ligand 2 (PD-L1 and PD-L2) has been demonstrated to have a critical role in dampening T cell responses to viruses and tumors. Chronic stimulation with persistent antigens results in the exhaustion of CD8^+^ T cells and PD-1, which is constitutively expressed by these cells, significantly contributes to their impaired function^17–20^. In addition to PD-1, T cells can express serval other coinhibitory receptors like CTLA-4 (cytotoxic T lymphocyte associated protein 4), BTLA (B- and T lymphocyte attenuator) and LAG-3 (lymphocyte activation gene 3). CTLA-4 and PD-1 pathways are currently targeted to enhance anti-tumor responses in melanoma patients and individuals suffering from various other cancers. BTLA and LAG-3 are emerging targets in cancer or infectious diseases^21,22^.

Importantly, the response of T cells is broadly controlled by inhibitory receptors whose presence is not limited to cells that have reached a state of exhaustion. Studies in animal models have highlighted the importance of T cell checkpoints in maintaining tolerance and preventing autoimmunity^23–25^. A role of these molecules in preventing immune pathologies was corroborated with the introduction of antibodies targeting coinhibitory pathways, so-called immune checkpoint inhibitors in the clinic: administration of PD-1 or CTLA-4 antibodies is associated with a large spectrum of side effects referred to as immune-related adverse events (irAEs)^26,27^. Moreover, it has been established that mutations in the human *CTLA4* and *PDCD1* loci are associated with various autoimmune diseases. Importantly some SNPs in these loci appear to be also linked with atopy as they were shown to be associated with IgE-levels, bronchial hyperresponsiveness and allergic asthma^28,29^. Studies in murine models indicate an important role of PD-1 pathways in asthma and demonstrate that PD-1 and BTLA are required for termination of acute allergic airway inflammation^30–32^. Taken together, these observations suggest that dysregulation of T cell inhibitory pathways can contribute to aberrant T cell responses resulting in autoimmunity and immune pathologies like IgE-mediated allergies.

Nonetheless, still little is known regarding the role of immune checkpoints in regulating allergen-specific human T cells. Here we have analyzed the expression of major immune checkpoints (CTLA-4, PD-1, BTLA and LAG-3) on T cells responding to common allergen sources. In addition, we have employed blocking antibodies to investigate the capacity of these receptors to inhibit allergen-specific CD4^+^ T cells derived from allergic as well as non-allergic individuals. Our results indicate a singular role of PD-1 in dampening allergen-specific human T cells.

## Results

### PD-1 engagement inhibits allergen-specific T cell clones

**Figure 1:**
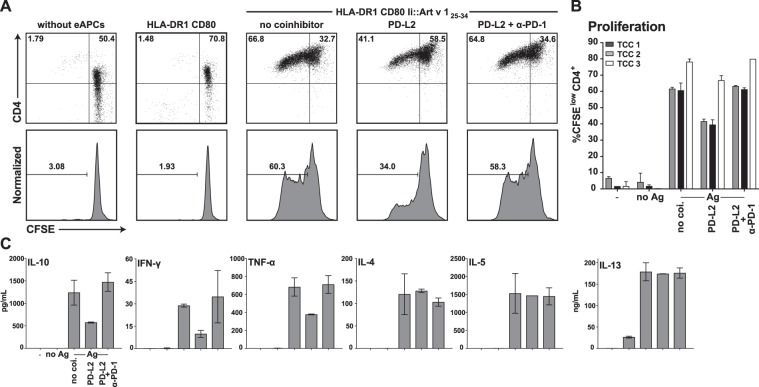
PD-1 coinhibition in allergen-specific CD4^+^ TCC using engineered APCs. (**A**) An Art v 1 specific T cell clone was CFSE-labeled and stimulated with cognate allergenic peptide Art v 1_25–34_ presented by engineered APC (HLA-DR1^+^CD80^+^) in presence or absence of PD-1 ligands (PD-L2). Cells were harvested, stained for CD4 and analyzed by flow cytometry after 4 days of cocultivation. PD-1 inhibition was disrupted using a functional grade PD-1 blocking antibody as indicated. (**B**) Summarized data of experiments performed as described in (**A**) using three Art v 1-specfic TCC. Experiments were performed in duplicates and the percentage of CFSE^low^ CD4^+^ T cells is shown as mean and SD. (**C**) Culture supernatants from (**A**) were collected and analyzed using Luminex. The content of IL-10, the T_H_1 cytokines IFN-γ and TNF-α as well as the T_H_2 cytokines IL-4, IL-5 and IL-13 is shown as mean and SD from duplicate wells. The cytokine data are representative for experiments with three different TCC.

In a first set of experiments we sought to assess whether engagement of immune checkpoints can inhibit allergen-specific T cell clones (TCC). To address this, we used a previously described engineered APC (eAPC) system based on HLA-DR1^+^ CD80^+^ K562 cells expressing the single dominant epitope of the major mugwort allergen Art v 1 (Art v 1_25–34_) targeted to the MHC class II presentation pathway via fusion to the invariant chain^33^. eAPC expressing the PD-1 ligand PD-L2 induced significantly lower proliferative responses in Art v 1 specific TCC compared to eAPC presenting Art v 1 in absence of PD-1 ligands and the presence of a PD-1 blocking antibody reverted this effect (Fig. 1A,B). To evaluate the influence of coinhibition blockade on cytokine production, supernatants of the TCC assays were analyzed for IL-10, the T_H_1 cytokines IFN-γ and TNF-α as well as the T_H_2 cytokines IL-4, IL-5 and IL-13 (Fig. 1C). Interestingly, PD-1 engagement reduced production of IL-10, IFN-γ and TNF-α, while IL-4, IL-5 and IL-13 were unaffected.

### CD4^+^ T cell responses of allergic and non-allergic individuals to common allergen sources

**Figure 2:**
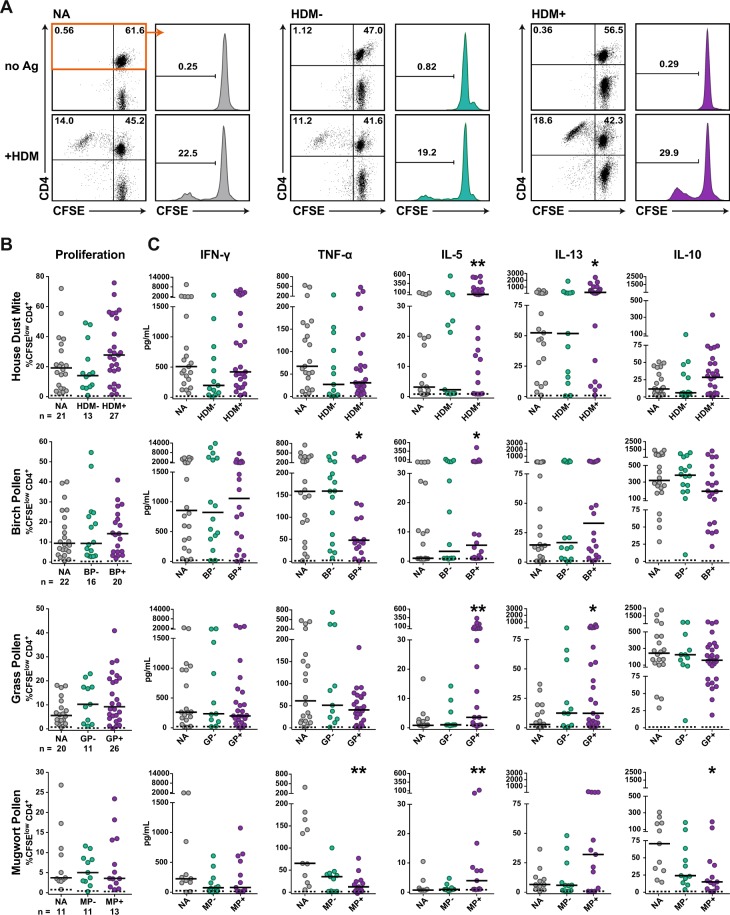
CD4^+^ T cell responses of allergic and non-allergic donors to common allergen sources. CFSE-labeled PBMCs of allergic and non-allergic individuals were stimulated with allergenic extract of house dust mites (HDM), birch pollen (BP), grass pollen (GP) or mugwort pollen (MP) for 6-7 days. Cells were harvested, stained for CD4 and analyzed by flow cytometry. (**A**) T cell proliferation of a representative non-allergic individual (NA), an allergic patient not sensitized to house dust mite (HDM−) and a house dust mite allergic patient (HDM+) in response to house dust mite extract is shown. Dot plots depict CFSE versus CD4 in live cells and histograms show percentage of CFSE^low^ cells gated on live CD4^+^ T cells. (**B**) Percentage of CFSE^low^ CD4^+^ T cells are shown for the indicated number of donors and median percentage of CFSE^low^ CD4^+^ T cells for each group is indicated as line. (**C**) Culture supernatants were collected, pooled from triplicates and analyzed by multiplex flow cytometry. Median concentration is indicated as line. (B,C) Dashed lines indicate data for unstimulated conditions. Statistical significances of differences in the responses to allergen stimulation between PBMCs derived from NA and allergic patients were calculated using the Mann-Whitney test (*P ≤ 0.05 and **P ≤ 0.01).

To assess the role of immune checkpoints in allergen-specific T cell responses, peripheral blood mononuclear cells (PBMCs) were obtained from non-allergic healthy donors (n = 23) and allergic donors (n = 41) suffering from inhalant allergies to birch pollen (BP), grass pollen (GP), mugwort pollen (MP) and house dust mites (HDM), see Table 1. PBMCs of allergic patients and healthy controls were CFSE-labeled and stimulated with allergenic extracts for HDM, BP, GP or MP for 6–7 days. Subsequently CD4^+^ T cell proliferation was measured by flow cytometry and the culture supernatants were subjected to multiplex cytokine analysis (LEGENDplex T_H_1/2 cytokine panel). For each stimulation condition, cultures were categorized into three groups: PBMCs derived from non-allergic donors (NA) and PBMCs from allergic donors not sensitized (−) or sensitized (+) to the allergens contained in the respective extract (Fig. 2A). There was a tendency of higher CD4^+^ T cell proliferation (Fig. 2B) and T_H_2 cytokine production in samples of allergic donors sensitized to the allergenic extract used for stimulation compared to samples of non-allergic donors (Fig. 2C). All allergenic extracts tested induced significantly higher IL-5 amounts in PBMCs of allergic donors compared to non-allergic donors, whereas IL-13 values were significantly higher only upon stimulation with extracts from house dust mites and grass pollen. By contrast, PBMC cultures of allergic donors stimulated with pollen extracts from birch and mugwort contained significantly less TNF-α than non-allergic donors (Fig. 2C). The majority of samples contained IL-2 and IL-4 values below the detection limit and low levels of IL-9, IL-17A, IL-21 and IL-22 (data not shown). Overall, our results demonstrate CD4^+^ T cell proliferation and cytokine production to allergen sources in the large majority of PBMC samples tested and a tendency towards stronger and more T_H_2-biased responses in samples derived from allergic donors. We observed a weak positive correlation of the specific IgE levels and CD4^+^ T cell proliferation in response to HDM and GP extracts but not to BP extract (Fig. S1). Moreover, our data illustrate qualitative differences in the responses to different allergen sources, for instance it was found that although HDM extract caused higher CD4^+^ T cell proliferation than BP or GP extract, its capacity to induce production of IL-10 was much lower (Fig. 2).

**Table 1:** Characteristics of the study cohort – Multisensitzed allergic patients were subgrouped according to their reaction profile.

Group	Sub-Group	Count	Sex (f/m)	Age (mean)	Age (range)	Total IgE (mean)	Total IgE (range)
**NA**		23	16/7	32.27	40.19	47.17	144.00
**A**		41	18/23	31.35	37.13	403.59	4995.58
	**HDM**	27	10/17	28.21	33.05	555.73	14393.83
	**BP**	23	10/13	29.19	32.63	632.34	1452.95
	**GP**	29	12/17	31.12	37.13	549.18	1275.84
	**MP**	14	9/5	28.76	33.05	944.55	1789.68

NA, non-allergic; A, allergic; HDM, house dust mite; BP, birch pollen; GP, grass pollen; MP, mugwort pollen.

### Expression of the immune checkpoints PD-1, LAG-3, BTLA and CTLA-4 on CD4^+^ T cells in response to common allergen sources

**Figure 3:**
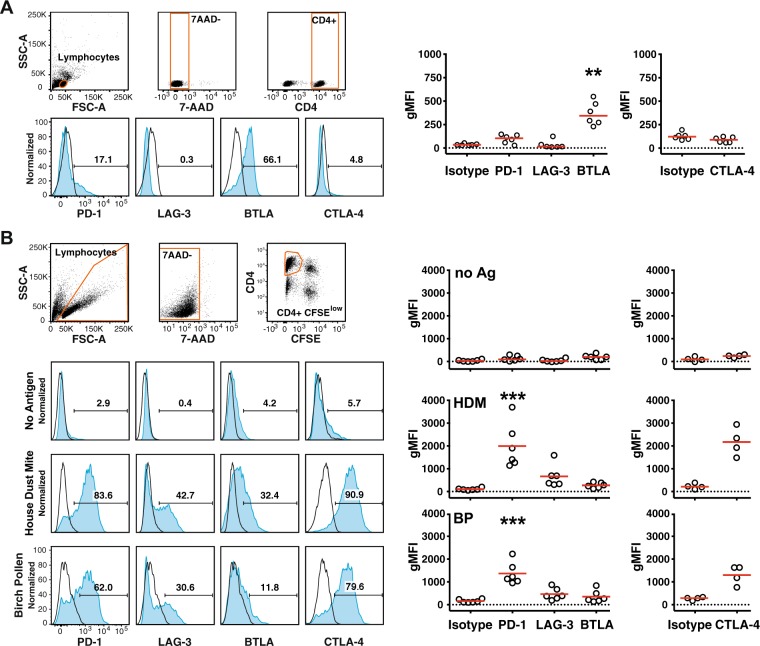
Expression of the immune checkpoints PD-1, LAG-3, BTLA and CTLA-4 by CD4^+^ T cells responding to common allergen sources. (**A**) Unstimulated PBMCs of study donors were analyzed for expression of indicated immune checkpoints using the gating strategy depicted in the upper panels. Blue histograms show the expression of indicated immune checkpoints of a representative allergic donor and numbers indicate percent receptor positive cells. Scatter plots on the right side show reactivity of antibodies to PD-1, LAG-3, BTLA and CTLA-4 and isotype control of 6 donors (3 NA, 3A). (**B**) CFSE-labeled PBMCs of four to six donors (2 NA, 4A) were left unstimulated or stimulated with allergenic extracts for 7 days. 7-AAD negative CFSE^low^ CD4^+^ T lymphocytes were analyzed for the expression of the indicated immune checkpoints as shown in the blue histograms for one representative donor (left) and summarized in cumulative scatter plots (right). For expression analysis in unstimulated CD4^+^ T cells the gate was set to CFSE^high^ cells. Open histograms represent isotype control stainings. gMFI; geometric mean fluorescence intensity. Statistical analysis was performed using the Friedman test (**P ≤ 0.01 and ***P ≤ 0.001).

We analyzed the expression of PD-1, LAG-3, BTLA and CTLA-4 in freshly isolated CD4^+^ T cells (Fig. 3A) and CD4^+^ T cells that proliferated in response to HDM or BP extracts (Fig. 3B). Our results showed in accordance with previous reports that the majority of freshly isolated T cells expressed BTLA^34–36^. In addition, a small subset of unstimulated CD4^+^ T cells expressed low levels of PD-1, whereas LAG-3 and CTLA-4 was not detected. When stimulating CFSE-labeled PBMCs with HDM or BP extract for seven days, strong upregulation of PD-1, LAG-3 and CTLA-4 was measured in CD4^+^ T cells that had proliferated. By contrast, BTLA was downregulated in CD4^+^ T cells upon stimulation (Fig. 3; Fig. S2).

### Impact of immune checkpoint inhibitors on CD4^+^ T cell proliferation in response to allergenic extracts

**Figure 4:**
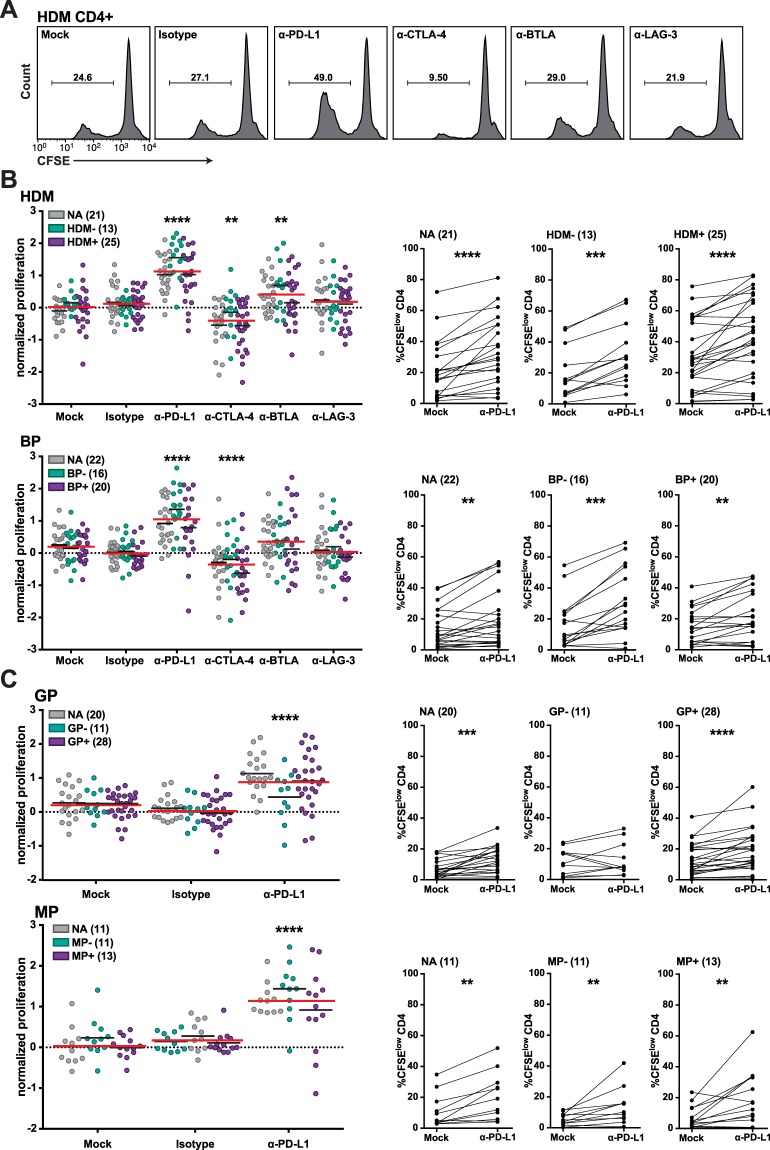
Influence of immune checkpoint inhibitors on CD4^+^ T cell proliferation in response to allergenic extracts. CFSE-labeled PBMCs of allergic and non-allergic individuals were stimulated with allergenic extracts in presence of blocking antibodies to the indicated molecules. After 6–7 days, cells were harvested, stained for CD4 and analyzed by flow cytometry. (**A**) Histograms show the effect of checkpoint inhibitors on CD4^+^ T cell proliferation of a representative HDM^+^ allergic patient in response to HDM extract. Numbers indicate percentage of CFSE^low^ cells gated on live CD4^+^ T cells. (**B**,**C**) Normalized proliferation scores and percentages of CFSE^low^ CD4^+^ T cells upon stimulation with HDM and BP extract (**B**) as well as GP and MP extract (**C**) in absence and presence of checkpoint inhibitors (left panels). Each data point represents the mean of triplicates of one donor. Values in brackets indicate numbers of donors. Median normalized proliferation for each group or the whole cohort is indicated as black and red line, respectively. Comparison of percentages of CFSE^low^ CD4^+^ T cells without and with PD-L1 blockade is depicted in the right panels. Stars indicate significant differences compared to mock control calculated with Friedman test and Dunn’s multiple comparison *post hoc* test (**P ≤ 0.01, ***P ≤ 0.001 and ****P ≤ 0.0001).

Immune checkpoint inhibitors were used to assess the role of PD-1, CTLA-4, BTLA or LAG-3 in CD4^+^ T cell responses to allergenic extracts. CFSE-labeled PBMCs of healthy controls and allergic individuals were stimulated with HDM or BP extracts (Fig. 4A,B). Blocking PD-1 signaling using a monoclonal PD-L1 antibody significantly increased CD4^+^ T cell proliferation from allergic patients as well as healthy individuals in response to all allergenic extracts tested (Fig. 4B,C). A blocking PD-1 antibody was similarly effective in enhancing T cell proliferation, whereas an antibody blocking PD-L2, the second PD-1 ligand, was not effective in enhancing T cell responses to HDM and BP (Fig. S3B,C). Antibodies to BTLA, LAG-3 and CTLA-4 were only tested in stimulation cultures with extracts of HDM and BP. BTLA blockade significantly enhanced CD4^+^ T cell proliferation in response to HDM extracts and there was a tendency for higher proliferation upon stimulation with BP extract. By contrast, LAG-3 blockade did not enhance CD4^+^ T cell proliferation in response to both extracts (Fig. 4B). Unexpectedly, the addition of the clinically applied CTLA-4 monoclonal antibody Ipilimumab profoundly and significantly reduced the CD4^+^ T cell response to allergenic extracts from HDM and BP. Earlier studies including work in our laboratory demonstrated that blocking additional immune checkpoints enhanced the effects of PD-1 blockade^35,37–40^. Therefore, we tested the capacity of antibodies blocking BTLA and LAG-3 to increase CD4^+^ T cell responses against house dust mite extract in combination with PD-L1 blockade. However, no synergistic effect of the analyzed inhibitor combinations was observed in these experiments (Fig. S3A).

### Effect of immune checkpoint inhibition on cytokine production in response to allergenic extracts

**Figure 5:**
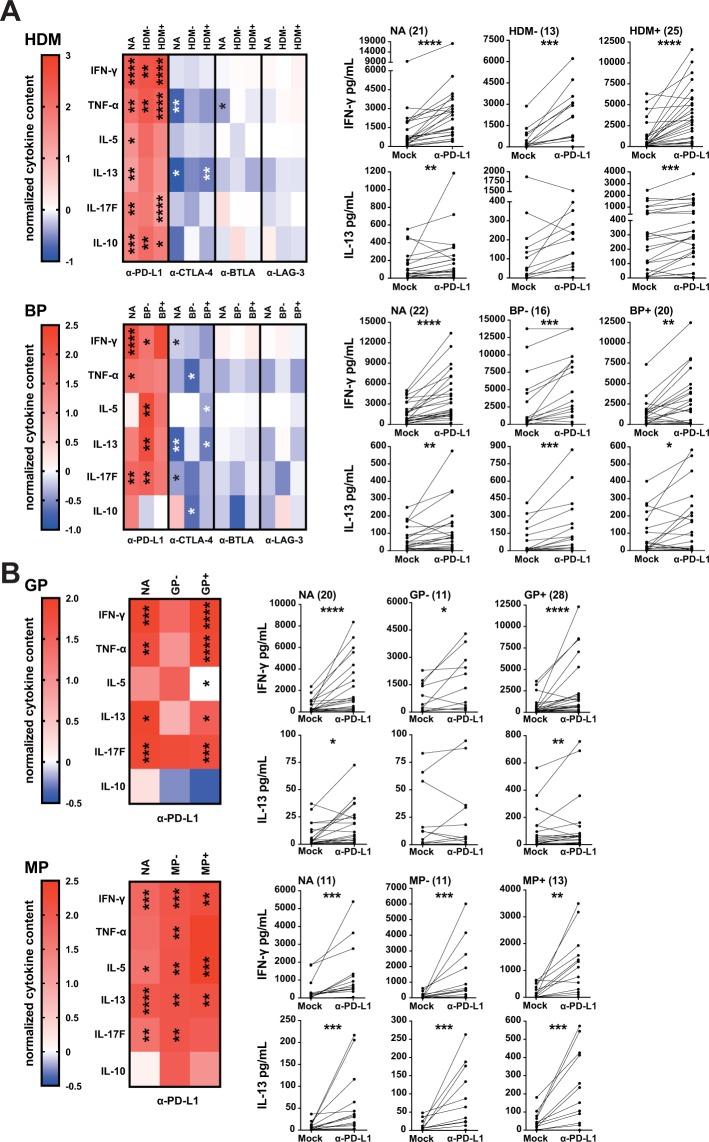
Influence of immune checkpoint inhibitors on the cytokine content in the culture supernatants in response to allergenic extracts. CFSE-labeled PBMCs of allergic and non-allergic individuals were stimulated with allergenic extracts of HDM and BP (**A**) as well as GP and MP (**B**) in the presence of blocking antibodies as indicated. After 6-7 days, culture supernatants were collected, pooled and analyzed by multiplex flow cytometry. (Left) Normalized cytokine scores (described in the material and methods section) of CD4^+^ T cells are summarized and shown in form of heat maps. The median values of IFN-γ, TNF-α, IL-5, IL-13, IL-17F and IL-10 are depicted for each cohort. (Right) Comparison of the total cytokine content of IFN-γ and IL-13 when stimulating PBMC cultures with the indicated antigen sources with or without PD-L1 blockade. Stars indicate significant differences compared to mock control calculated by Friedman test and Dunn’s multiple comparison *post hoc* test (*P ≤ 0.05, **P ≤ 0.01, ***P ≤ 0.001 and ****P ≤ 0.0001).

To evaluate the effect of immune checkpoint inhibitors on the production of cytokines by CD4^+^ T cells responding to stimulation, the concentration of 13T helper cell cytokines was determined in the supernatants using multiplex cytokine analysis. IL-2 and IL-4 were not detected and the low levels of IL-9, IL-17A, IL-21 and IL-22 in the samples were not significantly enhanced by immune checkpoint blockade (data not shown). By contrast, blocking PD-1 signaling potently enhanced production of T_H_1 (IFN-γ, TNF-α) and T_H_2 cytokines (IL-5, IL-13) as well as IL-17F in response to all allergenic extracts tested (Fig. 5A,B). IFN-γ and IL-13 production in response to allergenic extracts were both significantly increased in allergic and non-allergic donors. PD-L1 antibodies augmented the production of the inhibitory cytokine IL-10 to HDM extract, which was a low inducer of IL-10. By contrast, the response to extracts from BP and GP, which induced high levels of IL-10, was not affected by PD-L1 blockade (Fig. 5A,B). In line with the unexpected inhibition of CD4^+^ T cell responses upon addition of the CTLA-4 antibody Ipilimumab, we observed a trend towards reduced production of cytokines upon CTLA-4 blockade. This was most pronounced for TNF-α and IL-13 and did not reach statistical significance for all stimulation conditions (Fig. 5A). BTLA blockade, which had a positive impact on the proliferative responses, resulted in reduced production of cytokines with significantly lower TNF- α levels from non-allergic individuals stimulated with HDM extracts. LAG-3 blockade was ineffective in enhancing cytokine production in PBMCs of allergic and non-allergic donors in response to allergenic extracts (Fig. 5A). Together, our results point to a singular role of PD-1 blockade in enhancing CD4^+^ T cell responses to allergenic extracts.

## Discussion

IgE-mediated reactivity to proteins derived from HDM and pollen from birch tree, grass or mugwort are a major health problem in industrialized countries. It is well recognized that allergen-specific T_H_2 biased CD4^+^ T cell responses underlie this disease. Knowledge of the signals that orchestrate the T cell response to aeroallergens is thus mandatory to understand tolerance versus immunity towards exogenous antigens and may eventually aid in developing improved therapies for IgE-mediated diseases. Here, we have addressed the role of T cell expressed inhibitory receptors – often referred to as immune checkpoints – in the response to common sources of aeroallergens. Stimulation of PBMCs of allergic patients but also healthy individuals with protein extracts from HDM and pollen induced CD4^+^ T cell response in the majority of donors. Recent studies have highlighted that these responses are directed towards common allergens but also proteins that have low or no allergenic potential^15,41^. Moreover, also the phenotype of responding T cells is heterogonous and includes T_H_2 cells and non-T_H_2 effector cells. Within the T_H_2 population distinct subsets like terminally differentiated “T_H_2A” cells and T_H_2/17 cells have been proposed and implicated as drivers of allergic disorders and asthma, respectively^42,43^. In addition, a significant portion of regulatory T cells responding to aero-antigens have been reported in allergic as well as non-allergic individuals. Whereas earlier studies suggested that a balanced response of protective Tr1 and T_H_2 cells results in tolerance towards a given allergen^44,45^, work by Bacher and colleagues indicate that T_reg_ cells are common in non-allergic and allergic donors but are preferentially directed against non-allergens in the latter. The authors suggested that T_reg_ cells to a given aero-antigen actively maintain tolerance and preclude the development of high-avidity T_H_2 cells^41^. Thus, the T cell response against innocuous protein extracts is complex and interrelation between conventional T_H_ and T_reg_ response to common allergens and proteins with limited allergenicity is still incompletely understood. pMHCII tetramer based studies have yielded invaluable insight into the frequency, phenotype and function of CD4^+^ T cells recognizing major allergens^42,45–47^. However, the large number of allergens contained in sources like HDM or BP and MHCII diversity makes a comprehensive analysis of CD4^+^ T cell responses with this methodology difficult.

For our study, we thus have used protein extracts derived from common aeroallergen sources to stimulate CFSE-labeled PBMCs from allergic and non-allergic donors. The role of major inhibitory immune checkpoints (PD-1, CTLA-4, BTLA and LAG-3) on allergen-specific CD4^+^ T cells was addressed by the use of immune checkpoint inhibitors targeting these pathways. These antibodies were previously validated regarding their capacity to efficiently block the coinhibitory effects of their targets^35^. Blockade of PD-L1 strongly and significantly enhanced proliferation of CD4^+^ T cells and production of T_H_1 as well as T_H_2 cytokines in response to allergenic extracts (Figs 4 and 5). These effects were observed in PBMCs of allergic patients as well as those of healthy donors. In line, T cell clones that had been established from donors allergic to mugwort showed a reduced response to the major mugwort pollen allergen Art v 1 upon PD-1 engagement (Fig. 1). Blockade of BTLA, which was found to be constitutively expressed on CD4^+^ T cells and downregulated upon *in vitro* culturing, enhanced proliferation in response to HDM but was ineffective in increasing cytokine production response to allergenic extracts in all samples. Despite the presence of LAG-3 on CD4^+^ T cells that responded to HDM or BP extracts, blocking LAG-3 was ineffective in augmenting proliferation or cytokine production in response to these stimuli. This observation is in line with previous results obtained in our laboratory, which indicated that LAG-3 blockade does not augment the proliferation and cytokine production of T cells responding to HIV-1 antigens or allogeneic dendritic cells^35,37^. Interestingly, we found that the presence of the clinically used CTLA-4 monoclonal antibody Ipilimumab resulted in significantly reduced CD4^+^ T cell responses upon stimulation with extracts derived from HDM and BP (Figs 4A and 5A). In previous studies, we have observed a trend towards lower CD4^+^ T cell response upon presence of Ipilimumab, but this effect did not reach statistical significance in those datasets^35,37^. The mode of action of CTLA-4 antibodies is complex and incompletely understood. Recent studies suggested that *in vivo* CTLA-4 antibodies do not function by blocking CTLA-4 inhibition but by depleting T_reg_ cells in the tumor microenvironment via F_c_-receptor mediated mechanisms^48^. Romano and colleagues provided evidence that Ipilimumab-dependent cytotoxicity exerted by nonclassical monocytes mediates T_reg_ depletion in melanoma patients^49^. We show that CD4^+^ T cells responding to allergens strongly upregulate CTLA-4 and it is possible that F_c_-mediated depletion of conventional T cells contributes to surprising effects of the clinically used CTLA-4 antibody in our experiments.

Several studies including work in our laboratory have demonstrated that combined blockade of several immune checkpoints is more effective than blocking PD-1 alone^35,37–39^. When testing CD4^+^ T cell proliferation in response to HDM extracts we did not observe that combinations of immune checkpoint inhibitors were more effective than a PD-L1 antibody alone (Fig. S3A). In the clinic, PD-1 signaling is currently blocked by antibodies binding PD-1 or PD-L1. Expectedly, we have observed a similar effect of PD-1 and PD-L1 antibodies in our study, whereas a blocking antibody targeting the second ligands for PD-1, PD-L2, did not improve T cell responses (Fig. S3B,C). Several studies have suggested a role of PD-L2 in allergic responses, but contrasting effects were likewise described^50–52^. PD-L2 can also bind to the repulsive guidance molecule b (RGMb)^53^. This protein is highly expressed in the lung and its engagement promotes respiratory tolerance. Effects of PD-L2 antibodies on RGMb-PD-L2 interactions in the lung may be responsible for the different outcomes of PD-L2 and PD-L1 blockade in models of asthma and airway hyperreactivity.

To our knowledge, the study presented here is the first to compare the role of major T cell expressed immune checkpoints PD-1, CTLA-4, BTLA and LAG-3 in allergen-specific immune responses. Our results highlight a potent and singular role of PD-1 signaling in limiting CD4^+^ T cell responses towards aeroallergens. Releasing PD-1 inhibition dramatically enhances T cell proliferation and production of T_H_2 as well as T_H_1 cytokines. Although PD-1 function is frequently associated with CD8^+^ T cell responses towards virus and cancer antigens, the results presented here as well as several earlier studies underline that PD-1 signals can keep CD4^+^ T cells in check^35,54^. Thus, PD-1 agonists might be effective in limiting pathological CD4^+^ T cell responses in autoimmune diseases and allergies.

## Materials and Methods

### Patients, sample collection and cell isolation

Blood samples were collected from allergic individuals and healthy donors between 2014–2017 preferentially during the pollen seasons after signing a patient information and an informed consent. The study with primary human cells was approved by the ethics committee of the Medical University of Vienna (EK1538/2014) and procedures with human material were performed in accordance with the Helsinki Declaration of 1975. Diagnosis of allergic patients suffering from inhalant allergies (birch pollen, grass pollen, mugwort pollen and house dust mites) was based on a clinical history of typical symptoms like (allergic conjunctivitis, rhinitis and asthma) and a positive ImmunoCAP (Thermo Fischer Scientific Waltham, MA). Sensitization patterns were used to classify the allergic individuals (n = 41) as follows: house dust mite positive (HDM+, n = 27), house dust mite negative (HDM−, n = 14), birch pollen positive (BP+, n = 23), birch pollen negative (BP−, n = 18), grass pollen positive (GP+, n = 29), grass pollen negative (GP−, n = 12), mugwort pollen positive (MP+, n = 14) and mugwort pollen negative (MP−, n = 27), respectively. The control group (NA) consists of blood samples from 23 non-allergic healthy donors without allergic symptoms and devoid of IgE reactivity towards HDM, BP, GP and MP as confirmed by CAP. Epidemiological and clinical characteristics of the donors enrolled in the study are summarized in Table 1. Isolation of peripheral mononuclear cells (PBMCs) was performed from heparinized whole blood samples with standard gradient density centrifugation using Lymphoprep solution (Technoclone, Austria).

### CFSE proliferation assay

Pollen from birch tree (*Betula pendula*), grass (*Phleum pratense*), mugwort (*Artemisia vulgaris*) as well as house dust mites (*Dermatophagoides pteronyssinus*) were derived from Allergon (Thermo Fisher Scientific, Sweden) and preparation of allergenic extract was performed as described elsewhere^55^. Labeling of 1 × 10^7^ PBMCs was performed using 1 μL of a 1 mM CFSE stock solution (Molecular Probes) in 1 mL PBS for 4 min at room temperature. Subsequently after washing the cells, 1 × 10^5^ PBMCs were stimulated with allergenic extracts (40 µg total protein/mL) for 6–7 days in presence or absence of immune-checkpoint blocking antibodies (8 µg/mL) in 96 well plates. Percentage of CFSE^low^ CD4^+^ T lymphocytes was determined using flow cytometry. Mean of triplicates of one donor is shown as single data point. A stimulation index was calculated by dividing %pos CD4^+^ T cells of extract stimulated wells with %pos CD4^+^ T cells of unstimulated wells. Responses with an index of 1.5 or greater were considered positive and included in further analysis.

### Cell culture, antibodies and flow cytometry

The following monoclonal antibodies were used to evaluate surface expression on PBMCs and allergen-specific TCC: PD-1-PE (EH12.2H7), BTLA-PE (MIH26), CD4-PE (OKT4) and appropriate isotype controls (MOPC-21) all purchased from Biolegend (San Diego, CA). LAG-3-PE (3DS223H) and CTLA-4-PE (14D3) were purchased from eBioscience (San Diego, CA). Cells were stained in FACS buffer (PBS, 1% BSA, 0.1% NaN_3_) for 30 min. For immune checkpoint stainings, 10 mg/mL Beriglobin (CSL Behring) were added to prevent unspecific binding to F_c_-receptors. Exclusion of dead cells from analysis was done using 7-AAD (Biolegend) where appropriate. Intracellular CTLA-4 expression was determined using the Cytofix/Cytoperm kit from BD Biosciences according to the manufacturer’s instructions.

To assess the effect of coinhibitory pathways, the following monoclonal blocking antibodies were added at a final concentration of 8 μg/ml: functional grade PD-1 (EH12.2H7; LEAF), PD-L1 (29E.2A3; LEAF) and PD-L2 (MIH18; LEAF) from Biolegend, CTLA-4 (Ipilimumab, Yervoy), BTLA, LAG-3 and a mouse IgG1 κ isotype control antibody (MOPC-21; LEAF, Biolegend). Blocking antibodies to BTLA and LAG-3 was described previously^35^. Flow cytometry analysis was performed using FACSCalibur and LSRFortessa flow cytometers (BD Bioscience). FlowJo software (version 10.0.6., Tree Star, Ashland, OR) was used for data analysis.

### Cytokine analysis with LUMINEX and LEGENDplex

Supernatants of the TCC proliferation assays were harvested at day 5–6 and pooled from duplicate wells. IL-10, IFN-γ, TNF-α, IL-4 and IL-13 were measured with the Luminex 100 system (Luminex Inc., Austin, TX) according to the manufacturer’s instructions. Supernatants of the PBMC proliferation assays were harvested at day 6–7, pooled from triplicate wells and stored at −20 °C. IL-5, IL-10, IL-13, IL-17F, IFN-γ and TNF-α were determined using the LEGENDplex human T_H_ 1/2 cytokine panel (13-plex, Biolegend) according to the manufacturer’s instructions. Cytokine content of each donor is represented by a single dot.

### Stimulation of allergen specific T cell clones using eAPCs

Allergen specific T cell clones (TCC) were generated from PBMCs of allergic donors and labelled with 1 μL of 1 mM CFSE (Molecular probes). Engineered antigen presenting cells based on the K562 cell line were generated and equipped with HLA-DR1, CD80 and a fusion protein of the major mugwort pollen T cell epitope Art v 1_25–34_ as previously described^33^. TCCs were stimulated with 3 × 10^5^ irradiated eAPCs (90 Gy) in presence or absence of the coinhibitory ligand PD-L2 for 4–5 days. To block coinhibition functional grade LEAF PD-1 antibody was used. Proliferation was assessed by flow cytometry and all experiments were performed in duplicate.

### Statistics

Normalization of data (proliferation, cytokines) was performed to generate an arbitrary scale adapted from the standard score using the formula x_norm_ = (x-baseline)/SD. Baseline stands for the median percentage of CFSE^low^ CD4^+^ T cells of the control samples or concentration of cytokine content (allergenic extract stimulation cultures devoid of immune checkpoint inhibitors). SD represents the standard deviation of the proliferation/cytokine values of all extract stimulation conditions of a certain T cell donor. Thereby, the number of standard deviations by which the data point diverges from the respective control replicates is depicted as the normalized proliferation or likewise normalized cytokine content. Graphpad Prism 7 (GraphPad Software, Inc., La Jolla, CA) was used to perform statistical analyses. Mann-Whitney test and a non-parametric repeated measurement ANOVA (Friedman test) was performed to analyze proliferation and cytokine data. Comparison of the immune checkpoint conditions with the no antibody control condition was accomplished using Dunn’s multiple comparison post hoc test. *P* values under 0.05 were considered significant (*), *P* ≤ 0.01, (**), *P* ≤ 0.001, ^(^***^)^, *P* ≤ 0.0001, (****).

## Electronic supplementary material

Supplementary Information
