# Supplementary material for: PD-1 has a unique capacity to inhibit allergen-specific human CD4+ T cell responses
Source: Sci Rep. 2018 Sep 10;8:13543. doi: 10.1038/s41598-018-31757-z (PMC6131174; doi:10.1038/s41598-018-31757-z)
Supplement: Supplementary file 1 — Supplementary Information [file 41598_2018_31757_MOESM1_ESM.pdf]

SUPPLEMENTARY INFORMATION

PD-1 has a unique capacity to inhibit allergen-specific human CD4$^+$ T cell responses

Sandra Rosskopf[1], Beatrice Jahn-Schmid[2], Klaus Schmetterer[3], Gerhard J. Zlabinger[4] and Peter Steinberger[1]*

[1]Division of Immune Receptors and T Cell Activation, Institute of Immunology, Center for Pathophysiology, Infectiology and Immunology, Medical University of Vienna, Vienna, Austria. [2]Institute of Pathophysiology and Allergy Research, Center for Pathophysiology, Infectiology and Immunology, Medical University of Vienna, Vienna, Austria. [3]Department of Laboratory Medicine, Medical University of Vienna, Vienna, Austria. [4]Division of Clinical and Experimental Immunology, Institute of Immunology, Center for Pathophysiology, Infectiology and Immunology, Medical University of Vienna, Vienna, Austria.

*Address correspondence: Peter Steinberger, Institute of Immunology, Center for Pathophysiology, Infectiology and Immunology, Medical University of Vienna, Lazarettgasse 19, Vienna, Austria, Phone 0043-1-40160-33241, e-mail: peter.steinberger@meduniwien.ac.at

Supplementary Figures

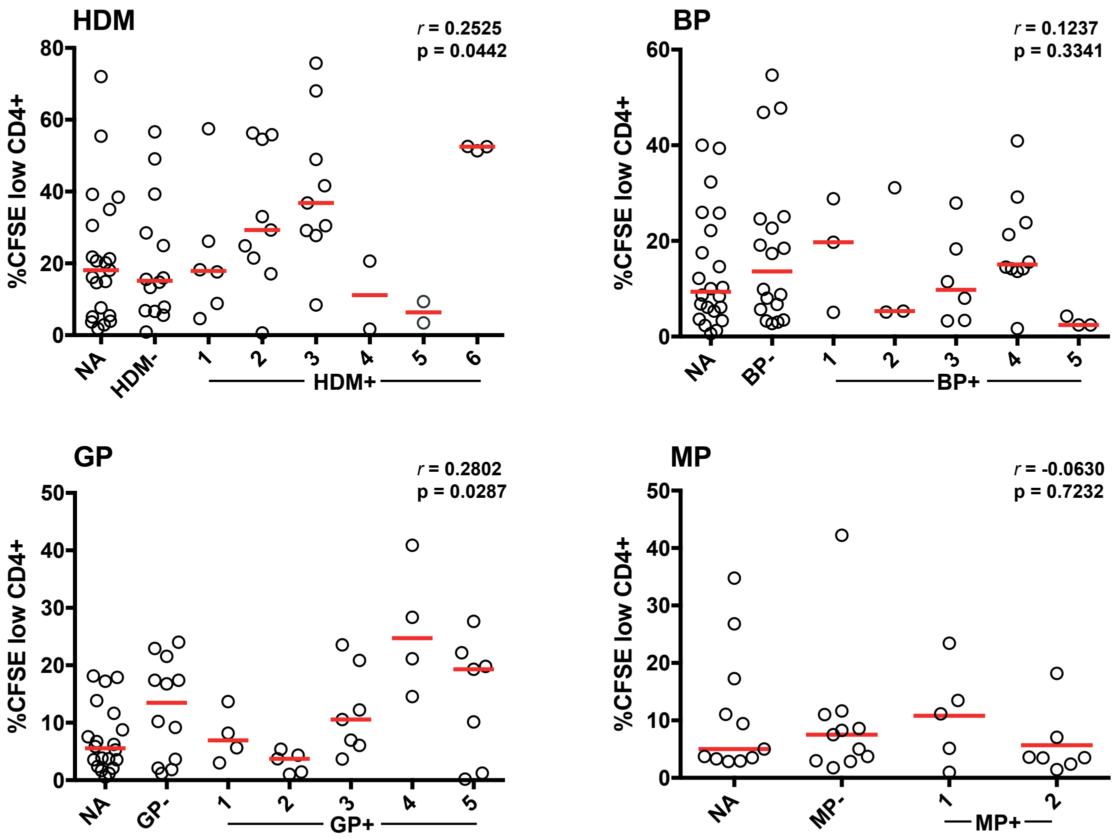

Supplementary Figure 1*: Correlation of T cell responses and CAP class*

CFSE-labeled PBMCs of allergic and non-allergic individuals were stimulated with allergenic extracts (HDM, BP, GP, MP) for 6-7 days. Cells were harvested, stained for CD4 and analyzed by flow cytometry. Percentage of $CFSE^{low}$ $CD4^{+}$ T cells of the study donors are shown in relation to CAP class measured after blood samples were taken. Each data point represents the mean of triplicates of one donor and median percentage of $CFSE^{low}$ $CD4^{+}$ T cells for each group is indicated as red line. Statistical analysis was performed using nonparametric Spearman correlation (*P ≤ 0.05).

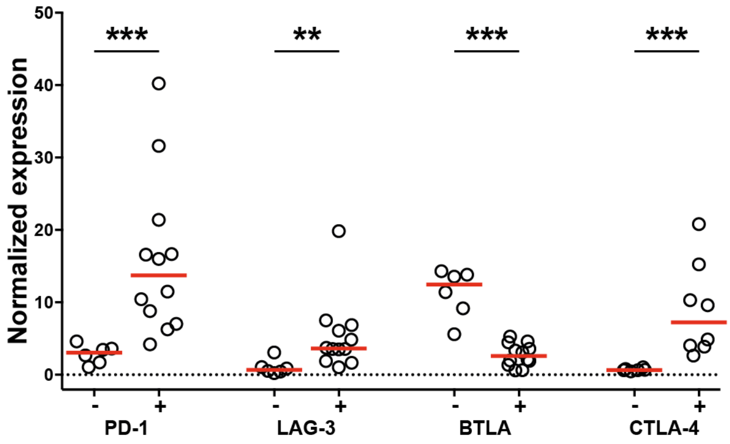

Supplementary Figure 2: *Expression of the immune checkpoints PD-1, LAG-3, BTLA and CTLA-4 in $CD4^{+}$ T responding to common allergen sources*

Data shown in Figure 3 was normalized to compare the expression of indicated immune checkpoints on unstimulated (-) as well as BP- and HDM-stimulated (+) $CD4^{+}$ T cells of study donors. Normalization was done by dividing gMFI values of stainings with antibodies to the indicated molecules divided by gMFI values of isotype control stainings. Statistical analysis was performed using Mann-Whitney tests (***P* ≤ 0.01 and ****P* ≤ 0.001).

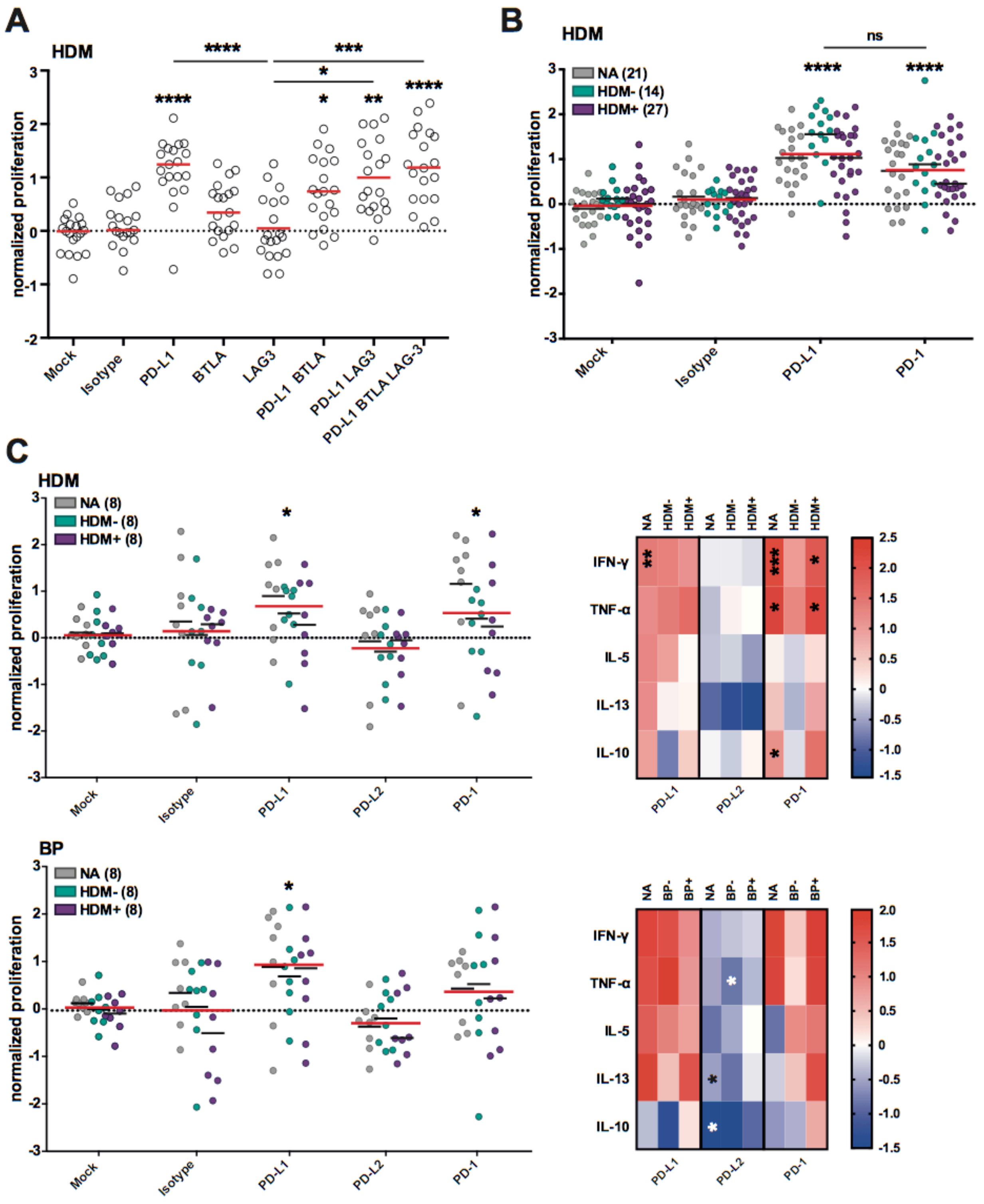

Supplementary Figure 3*: Influence of immune checkpoint inhibitors on CD4^{+} T cell proliferation*

CFSE-labeled PBMCs of allergic and non-allergic individuals were stimulated with indicated extracts in absence (mock) or presence of isotype control or blocking antibodies to indicated molecules for 6-7 days. Cells were harvested, stained for CD4 and analyzed by flow cytometry. Normalized proliferation scores of CD4^{+} T cells are shown and each data point represents the mean of triplicates of one donor. Median normalized proliferation for each group as well as the whole study cohort is indicated as black and red line, respectively. (A) Assessment of the combinatorial effect of immune checkpoints on T cell proliferation stimulated with HDM extract (10 NA, 4 HDM-, 5 HDM+). (B) Influence of PD-L1 and PD-1 blocking antibodies on CD4^{+} T cell proliferation upon HDM extract stimulation. (C) Effect of blocking antibodies to PD-1, PD-L1 and PD-L2 on the proliferation of CD4^{+} T cells stimulated with HDM or BP extract. Normalized proliferation scores (left) and normalized cytokine contents (right) in absence or presence of indicated antibodies are shown. Statistical analysis was performed using Friedman test and Dunn's multiple comparison *post hoc* test in comparison to mock control and amongst the groups (*P ≤ 0.05, **P ≤ 0.01, ***P ≤ 0.001, ****P ≤ 0.0001, ns … not significant).
